# Inheritance and QTL analysis of the determinants of flower color in tetraploid cut roses

**DOI:** 10.1007/s11032-016-0565-9

**Published:** 2016-10-07

**Authors:** Virginia W. Gitonga, Robert Stolker, Carole F. S. Koning-Boucoiran, Mitra Aelaei, Richard G. F. Visser, Chris Maliepaard, Frans A. Krens

**Affiliations:** 1 Wageningen UR Plant Breeding, P.O. Box 386, 6700 AJ Wageningen, The Netherlands; 2 Department of Horticulture, Tehran University, Karaj, 31587-77871 Iran; 3 Selecta Kenya GmbH & Co. KG, P.O. Box 64132, Nairobi, 00620 Kenya; 4 Deliflor Chrysanten b.v., P.O. Box 77, 2676 ZH Maasdijk, The Netherlands; 5 HAN University of Applied Sciences, P.O. Box 6960, 6503 GL Nijmegen, The Netherlands; 6 Department of Horticultural Sciences, University of Zanjan, Zanjan, 45371-38791 Iran

**Keywords:** Tetraploid rose, Color determinants, Inheritance, QTL analysis, Genetics, Rosa × hybrida

## Abstract

The success of cut rose cultivars is a direct result of their aesthetic value. The rose industry thrives on novelty, and the production of novel flower color has been extensively studied. The most popular color is red, and it is, therefore, important for breeders to produce a good red cultivar. The final visible color of the flower is a combination of a number of factors including the type of anthocyanin accumulating, modifications to the anthocyanidin molecule, co-pigmentation and vacuolar pH. Here, we analyze the quantitative variation of the biochemical constituents of flower color in a tetraploid rose population and combine this with marker information in the segregating rose population to map the chromosomal locations of putative QTLs for flower color traits. Within our tetraploid population, we found a number of QTLs that were mapped on ICM 1, 2, 6 and 7. We were able to show the effect of the different QTLs on the final visible color of the flower from salmon to dark red.

**Electronic supplementary material:**

The online version of this article (doi:10.1007/s11032-016-0565-9) contains supplementary material, which is available to authorized users.

## Introduction

*Rosa* is the most important genus of ornamental horticulture, economically. In 2013, the Netherlands exported over 3.3 billion cut rose stems worth over 1 billion Euro (CBS 2013; Czechowski et al. 2004). The genus *Rosa* belongs to the family of the Rosaceae. While the genus *Rosa* comprises more than 150 species and thousands of cultivars (Gudin 2000), only 11 species were used to create the modern rose we know today (Crespel et al. 2002). The rose industry thrives on novelty, and the production of novel flower color has been studied extensively. Flower color results from the preferential absorption of part of the visible light by one or several chemical compounds (pigments) synthesized in the plant. Color is predominantly due to three types of pigments: flavonoids, carotenoids and betalains. The flavonoids are the most common of the three types of pigment and contribute to a range of colors from yellow to red to blue. The final visible color of the flower is a combination of a number of factors including the type and amount of anthocyanins accumulating, e.g., cyanin and/or pelargonin, modifications to the anthocyanidin basic molecule, e.g., glycosylation (type and location of sugar moiety), co-pigmentation and vacuolar pH (Brouillard 1988). In general, anthocyanidins are stabilized by glucosylation as anthocyanidin-3-*O*-glucosides, called anthocyanins; in roses, however, anthocyanidins are mostly glucosylated at two positions as 3,5-*O*-diglucosides which are then referred to as anthocyanins (Ogata et al. 2005). Each of these factors is regulated by a number of genes (Holton and Cornish 1995).

### The flavonoid pathway

The flavonoid pathway that results in production of anthocyanins is generally conserved among plant species and is well studied and documented (Holton and Cornish 1995; Tanaka et al. 1998). Rose pigments are composed mainly of anthocyanins (Jay et al. 2003). These anthocyanins are extremely sensitive to their microenvironment. For example, changes in pH as well as changes in concentration of metals and co-pigments (Brouillard et al. 1990) may all cause a shift in the absorption spectra of the anthocyanins. This may lead to a change in the reflective color of the flowers. Environmental factors such as light and temperature also influence pigmentation. In roses, low temperatures cause high concentrations of pigment (Biran and Halevy 1974) and a decrease in light intensity leads to a decrease in pigmentation.

Flavonoid compounds are produced as a branch of the phenylpropanoid pathway, a major secondary pathway that exists in all higher plants. Flavonoids have a C6-C3-C6 skeleton structure, commonly consisting of two aromatic rings (A- and B-ring) and one heterocyclic ring (C-ring). Based on the hydroxylation pattern of the B-ring, three major basic anthocyanidins can be found in roses: pelargonidin (one hydroxyl group), cyanidin and peonidin (both two hydroxyl groups, in peonidin one is methylated), which contribute to orange to red, red to magenta and magenta to purple colors, respectively. (Forkmann and Martens 2001; Mikanagi et al. 2000).

The three main anthocyanidins found in roses, cyanidin, pelargonidin and peonidin, occur as 3,5-*O*-diglucosides of the respective pigments and are then referred to as cyanin, pelargonin and peonin (Marshall et al. 1983). Interactions of the pigments give rise to the numerous shades of color we see (Lammerts 1945).

Several studies have been performed to date to better understand the inheritance of color in roses. De Vries et al. (1974) found a normal distribution over the cyanin classes of 200 varieties, but the distribution over the pelargonin was not clear. He also concluded that cyanin was not dominant over pelargonin since both pigments follow their own pattern of inheritance. De Vries et al. (1980a) demonstrated that the inheritance of both pelargonin and cyanin is mainly controlled by additive gene action. Additive gene action means that each locus in a group of nonallelic genes has a specific value that it contributes to a polygenic trait (De Vries et al. 1974; De Vries et al. 1980a). De Vries et al. (1980a) made a large number of crosses between 18 varieties with a known pigment composition. They were able to show additive gene action for all pigments, since it was not possible to classify the pigment contents of the rose seedling populations into clearly separate groups. Marshall et al. (1983) examined the heritability of the three main anthocyanins in more than 1200 progeny from 47 families using mid-parent/offspring regression and showed that each of them was highly heritable (high narrow-sense heritability estimates) in each population and that they all showed quantitative inheritance. The segregation frequencies for cyanin showed peaks that varied with the mean of the parents. Debener and Mattiesch (1999) studied the inheritance of pink flower color in *R. multiflora* hybrid populations and found a monogenic or oligogenic inheritance, and subsequently mapped the gene for pink flower Blfa on LG2. In contrast to this, Henz et al. (2015) found that the phenotypic variance they observed indicated a quantitative inheritance of anthocyanin confirming the observations of Marshall et al. (1983). Shupert et al. (2004) examined the inheritance of white/pink flower color in roses and found that the pink flower color is controlled by a major codominant gene. Their backcross populations, however, showed variation in color intensity, suggesting that other factors are also involved in the pink flower color. Henz et al. (2015), within their diploid population, observed quantitative variation in the total anthocyanin content; they hypothesized that this may be due to special combinations of alleles in the parents that do not lead to homozygous recessive progeny for key genes in anthocyanin biosynthesis. They mapped two major QTLs, one on LG2 for F3H and one on LG6 for bHLH genes, and several minor QTLs on LG3 and LG4.

The objective of the present study is to study the inheritance of flower color and biochemical constituents of flower color in a tetraploid rose population and to combine this with marker information in the segregating rose population to map the chromosomal locations of putative QTLs for flower color traits.

## Materials and methods

### Plant material and environments

**Fig. 1:**
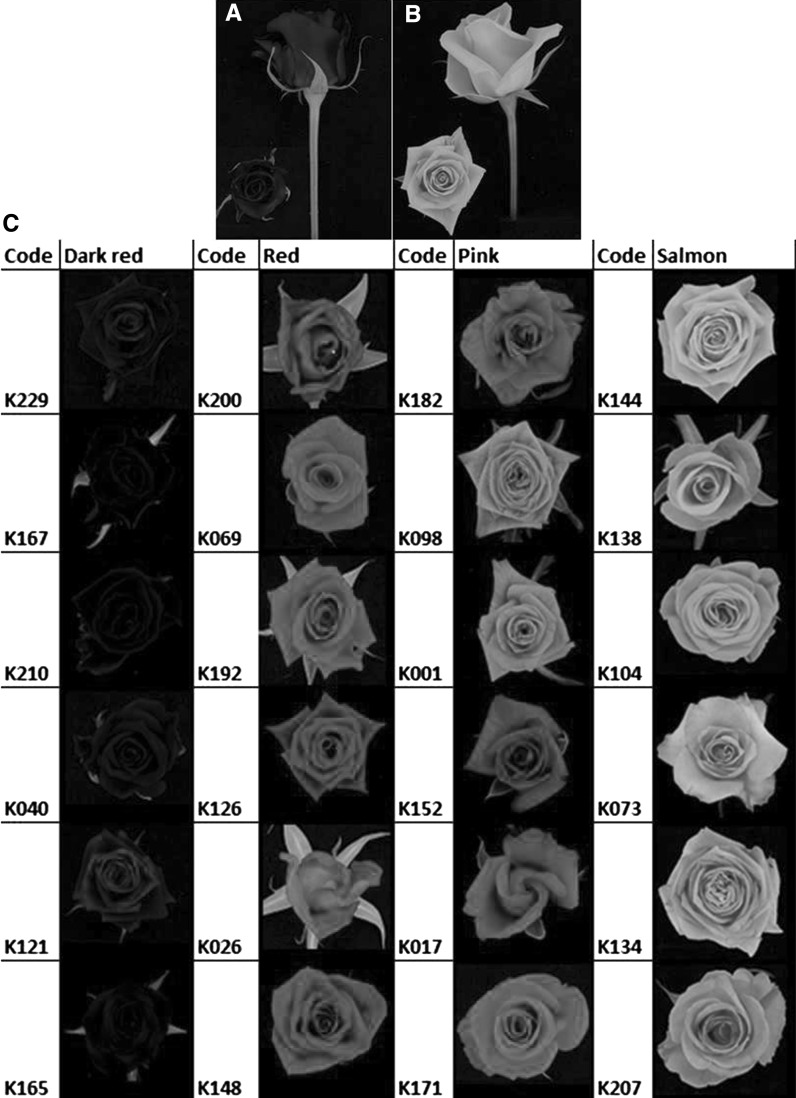
Range of flower colors in the K5 population (**c**) and the parents (**a**: P540, *red*; and **b**: P867, *salmon*). (Color figure online)

The K5 tetraploid rose population used in this study was described by Yan et al. (2005) and Koning-Boucoiran et al. (2012). This population, which originally was comprised of 184 genotypes, is the result of a cross between two tetraploid genotypes, P540 and P867 (Fig. 1a, b). The trial was established in Wageningen, The Netherlands **(**51°59′0″N, 5°40′0″E, 11 m altitude). The measurements were taken in the winter of 2007/2008.

Rooted nodal cuttings of each genotype, including the parents, were produced by Dümmen Orange (formerly known as Terra Nigra BV), a Dutch rose breeding company. The cuttings were planted in pairs in pots of coco peat in a greenhouse. The greenhouse was artificially lit to ensure a day length of 18 h. The temperature was kept at 20 °C (day temperature) and 17 °C (night temperature), and the relative humidity (RH) was kept between 80 and 90 %. A randomized complete block design was set up with two blocks and one replicate pot with two plants of the same genotype per block. In total, 129 genotypes were fully represented in the data presented here. Flowers, in developmental stage three (Fig. 1), were harvested from the greenhouse. From each genotype we had two biological replicates, one from each plot and we sampled two flowers per plant per genotype, so in total four flowers/genotype.

### Colorimetric measurements

#### Reflectometry

From each flower, the first three petals were discarded and from the remaining petals two petals were randomly selected and the inner and the outer epidermis were measured using a spectrocolorimeter (Spectrophotometer: Ocean Optics, Inc. SD2000 Lightsource: Top Sensor Systems, with halogen lightsource: HL 2000 FSHA Fitted: Bifurcated fiber: FCR-74V200-2-ME-S1White reference: Top Sensor Systems WS-2). The resulting reflectance curves show the percentage reflected light on every wavelength in the visible spectrum, representing a specific color. To be able to quantitatively describe the color, the curves were further translated into indices calculated using the L*a*b* color space developed by the *Commission Internationale d’Eclairage* (CIE) (in short: CIE L*a*b* color space). The values were calculated with reference standard observer of 10° visual field and reference to the standard illuminant D65 (daylight) at Δ*λ* of 10 nm. The L* parameter represents the luminance of the color ranging from 0 (black) to 100 (white). The a* value represents the position between red (positive values) and green (negative values). The b* parameter represents the position between yellow (positive values) and blue (negative values). From these coordinates, two more parameters are calculated: the hue angle (*h*° = arc tangent b*/a*) represents a basic color like red (~29°), orange (~45°) or yellow (~70°), while the chroma (C*) of the color represents the saturation of the color [C* = √(a*^2^ + b*^2^)] (Biolley and Jay 1993). A color with a high chroma looks very luminous and concentrated, while the same color with a low chroma looks dull, grayish and faded (keeping lightness and hue the same).

#### UV/Vis spectrometry

Depending on the size of the flower head, 6–9 petals were selected and immediately frozen in liquid nitrogen and kept at −80 °C in a freezer for anthocyanin analysis. The petals were further dried in a freeze dryer for 24 h and subsequently ground using a bead miller type MM2 from Retsch for 2 min at 50 % max RPM using 100 mg for dark and 200 mg for light colored rose samples. The ground rose samples were suspended in 5 mL of 1 % (v/v) hydrochloric acid (HCl) and 0.1 % (w/v) butyl hydroxyl anisol (BHA) in methanol for 30 min at room temperature with shaking at 150 RPM in a shaker type RS500 from LaboTech. The extracts were centrifuged and the process repeated until all the anthocyanin was extracted from the samples. The supernatant was filtered through a microfilter (0.45 μm) and used for spectrometry or HPLC. Spectroscopic analyses of the non-hydrolyzed anthocyanin extracts were carried out on a wavelength-scanning UV/Vis spectrophotometer (Ultrospec 2000, Pharmacia Biotech) controlled by SWIFT WAVESCAN II applications software. Cuvettes with a light path of 1 cm were obtained from Brand (Wertheim, Germany). The reference solution was 1 % (v/v) hydrochloric acid (HCl) and 0.1 % (w/v) butyl hydroxyl anisol (BHA) in methanol. The absorbance was recorded at 1 nm steps over the range 350–750 nm at a scan rate of 2500 nm min^−1^. The wavelengths of peak absorbance (*λ*_max_) were recorded.

#### HPLC

Complete acid hydrolysis was obtained from 2 ml volume of the sample solution with 2 ml of 2 N HCl at 100 °C after 2 h. Isolated, deglucosylated compounds, i.e., the anthocyanidins, were characterized by HPLC. Ten µL of the filtered supernatant were injected into a C18 reversed-phase column (3 µm particle size, 150 mm × 3 mm) from Thermo Scientific (Cheshire, UK) and was protected with a C18 HyPurity (5 µm particle size, 10 mm × 3 mm) drop-in guard precolumn from Thermo Scientific. The separation of anthocyanidins was done by using 10 min elution with 1.5 % (v/v) phosphoric acid in water, followed by 20 min linear gradient increase elution from 30 % to 50 % with 1.5 % (v/v) phosphoric acid and 20 % (v/v) acetic acid and 25 % (v/v) acetonitrile in water. The column chromatography was performed at 40 °C, and samples were eluted at a flow rate of 0.8 ml min-1. Anthocyanidins were monitored at 512 nm using a photodiode array detector.

Anthocyanidins detected in HPLC profiles were identified by matching their retention times to those of anthocyanidin standards (cyanidin chloride, pelargonidin chloride, peonidin chloride and delphinidin chloride) obtained from Sigma-Aldrich Chemie BV (Zwijndrecht, the Netherlands). The three different anthocyanidins found in roses, cyanidin chloride, pelargonidin chloride and peonidin chloride, and the fourth anthocyanidin, delphinidin chloride, elute at 15.3–15.5, 18.3–18.5, 19.7–19.9 and 12.1–12.2 min, respectively.

To quantify the peak areas, calibration plots for the different anthocyanidins were constructed at four concentration levels (10, 20, 40 and 100 mg L^−1^) of cyanidin chloride, pelargonidin chloride, peonidin chloride and delphinidin chloride and were analyzed against the peak area at 512 nm. The calibration curves for the anthocyanidins were obtained by plotting the anthocyanidin peak area against the anthocyanidin concentration at four levels. The calibration plots linearity was excellent, with regression coefficients of 0.9998, 0.9995, 0.9999 and 0.9999, respectively. Quantification of anthocyanidins was performed by correlating the chromatographic peak area with concentrations in accordance with the calibration plot of the corresponding external standard. The anthocyanidin concentrations were expressed in mg g^−1^ petal dry weight.

An unidentified anthocyanidin was found in the K5 population that was initially thought to be a product of incomplete hydrolysis of cyanin, but it showed a peak at 10.5–10.6 min which was outside the expected 15.3–15.5 min peak of cyanidin chloride.

### Phenotypic data analysis

Descriptive statistics per trait were calculated in Genstat 16 (GENSTAT 2013). Only two of the four major anthocyanidins, cyanidin and pelargonidin, and the unidentified anthocyanidin were present in the K5 population. For each of the color traits, an analysis of variance (ANOVA) was performed to estimate the means of the genotypes. Pearson correlation coefficients between the phenotypic traits were calculated as a measure of the strength of linear association using Genstat 16 (GENSTAT 2013).

### QTL mapping

#### Linkage map and QTL analysis

The parental maps of the P540 and P867 were constructed by Koning-Boucoiran et al. (2012) using AFLP, NBS and SSR molecular markers and later extended with SNP markers by (Koning-Boucoiran et al. 2015; Vukosavljev et al. 2015). Each chromosome was numbered 1–7 according to the integrated consensus map (ICM) by Spiller et al. (2011).

An analysis of variance (ANOVA) was conducted for each independent marker on dosage classes using an in house R script at a 99.9 % confidence level for each single test (*α* = 0.0001) on all the 15154 single nucleotide polymorphism (SNP) markers generated by Koning-Boucoiran et al. (2015) as described by Carvalho et al. (2015). Thereafter, we adjusted the *p* values using the Benjamini–Hochberg method (Benjamini and Hochberg 1995) and selected all the markers that had an adjusted *p* value of <0.05 and identified them to indicate QTLs.

## Results

### Color attributes

Within the K5 population, we observed a wide range of colors from dark red to very light salmon color (Fig. 1c). We observed that in all the flowers there was a difference in the visible color on the inner and outer sections of the flower. For each individual, the average colorimetric values for the inner and outer side of the petals were calculated. Table 1 shows an overview of the average, minimum and maximum L*, a*, b*, C*, *h*°, *λ*_max_, cyanidin, unidentified anthocyanidin and pelargonidin values for both the inner and outer side of the petal. The outer side of the petal is the visible side when the flower is still a bud, and the inner side is the part of the petal that can be seen only when the flower opens.

**Table 1:** Descriptive statistics of the color determinants of the parents (4 replicates per parent) and the K5 population

Trait	Parents		F1 progeny	
	P540 (dark red)	P867 (salmon)	K5 population	
	Mean ± SD	Mean ± SD	Mean ± SD	Range
L* Inner	41.3 ± 1.5	94.4 ± 1.5	60.4 ± 19.6	18.1–97.0
L* Outer	31.5 ± 3.1	93.5 ± 0.8	65.2 ± 15.2	36.3–97.8
a* Inner	41.5 ± 3.2	20.8 ± 3.7	62.9 ± 17.6	4.7–83.6
a* Outer	57.8 ± 3.2	26.8 ± 2.0	56.7 ± 14.5	8.9–74.7
b* Inner	46.7 ± 1.5	21.2 ± 8.7	41.1 ± 20.8	1.4–97.1
b* Outer	31.3 ± 4.1	23.3 ± 7.4	35.6 ± 17.5	3.6–70.3
C* Inner	62.4 ± 3.3	30.3 ± 6.9	76.7 ± 2.4	22.3–122.4
C* Outer	65.7 ± 4.8	35.9 ± 4.9	68.7 ± 16.7	21.6–98.9
*λ*_max_	524.0 ± 0.0	507.0 ± 3.2	521.9 ± 4.2	509.0–524.0
*h*° Inner	28.4 ± 0.5	51.8 ± 0.5	32.3 ± 12.2	1.4–82.5
*h*° Outer	48.4 ± 0.4	59.7 ± 0.3	31.5 ± 13.4	3.7–74.9
Cyanidin	17.2 ± 1.8	0.0 ± 0.0	4.9 ± 4.8	0.0–20.0
Pelargonidin	0.0 ± 0.0	0.4 ± 0.0	0.9 ± 2.2	0.0–16.9
Unidentified	0.7 ± 0.6	0.0 ± 0.0	0.2 ± 0.3	0.0–1.2

For the inner side of the petal, lightness (L*) ranged from 18 to 97 for darkest to lightest individual, for the outer side L* ranged from 36 to 97. Parent P540 had L* values of 41 and 32 for the inner and outer petals, parent P867 had 94 and 93 on the inner and outer petals, respectively. This indicates that within the F1 population, we had genotypes lighter and darker than the parents.

While the reflection measurements on the inner side of the petal resulted in an average a* and b* value of, respectively, 62.88 and 41.05, the outer side of the petal has an average a* and b* value of, respectively, 56.73 and 35.58. The chroma was calculated from a* and b* [C* = √(a*^2^ + b*^2^)]; the average C* of the inner side of the petal (76.66) was higher than the C* of the outer side of the petal (68.69). This means on average, the color on the inner side of the petal is more saturated and looks more luminous than the outer side of the petal. The chroma (C*), indicating the saturation of the color, varied from 122 for the most saturated individual with a pure and bright red flower color to 22 for an individual with a pale and impure (shades of light green) pinkish color. In general, the color of the outer side of the petal was lighter and less saturated than the inner side of the petal. The colors obtained from the calculated L*, a* and b* coordinates were in good agreement with the visual observation of flower color (Supplementary Fig. 1).

There was a wide range of hue angles (*h*°) exhibited by the K5 population. When the angle is close to 0° (or 360°), this indicates a magenta color with a blueing component. From 0° to 29°, the red shades contain less and less of the blueing component, resulting in a shift from purplish red to red. All individuals in the K5 population exhibit a hue within the range of 0°–90°, with 1° for the lowest and 82° for the individual with the highest hue (Table 1).

The individuals in the K5 population exhibited a *λ*_max_ ranging from 509 to 524 nm. The dark red parent P540 had a *λ*_max_ of 524 nm, and the salmon parent P867 had a *λ*_max_ of 507 nm.

Cyanidin and pelargonidin derivatives were the major pigments in the K5 population. Within the population, 98 % of the individuals were found to contain cyanidin in varying concentrations with the remaining 2 % of the progeny showing traces of cyanidin. Pelargonidin and the unidentified anthocyanidin were found in varying concentrations in, respectively, 35 and 43 % of the flowers. The unidentified anthocyanidin was confirmed not be cyanidin as in the HPLC analysis it was found to have a peak at 10.5–10.6 min which was outside the expected 15.3–15.5 min peak of cyanidin. Although highly correlated with cyanidin, the unidentified anthocyanidin was not a product of partial hydrolysis of cyanin. The highly positively correlated chemical variables, that are cyanidin and amount of unidentified anthocyanidin, were negatively correlated with the trait lightness (L*) of the petal color (Supplementary Table 1). The higher the concentration of cyanidin, the darker the flower (Supplementary Fig. 1). A very high concentration of cyanidin was seen in the individuals K210 and K167 (Fig. 1c), characterized by the darkest (L* = 18.1) and the second darkest color (L* = 19.4). They contained a high amount (20 and 12 mg g^−1^ petal dry wt., respectively) of cyanidin.

### QTL analysis

**Fig. 2:**
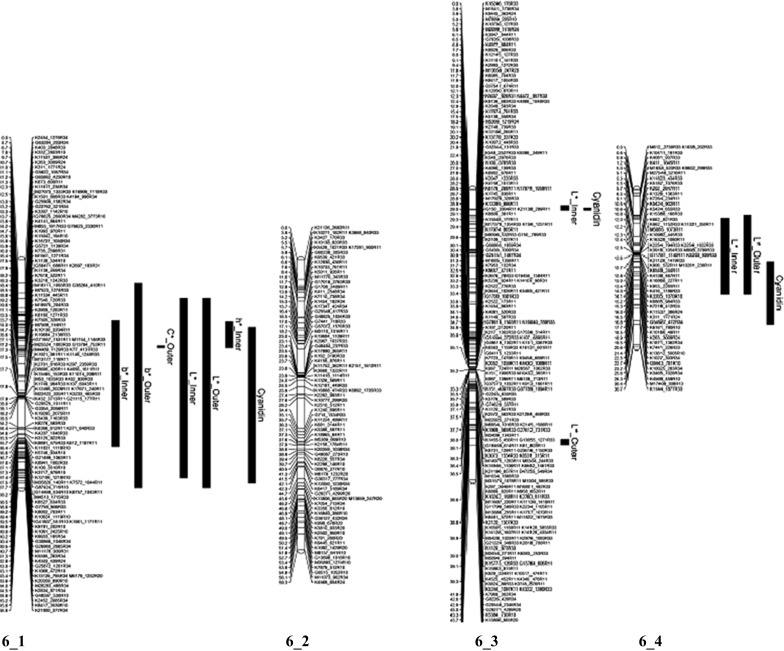
Four homolog linkage groups of ICM 6 of the genetic map of parent P540. The positions of the QTLs are represented by *black bars* (the *bar* presents the *lower* and *upper bounds* of the QTL)

**Fig. 3:**
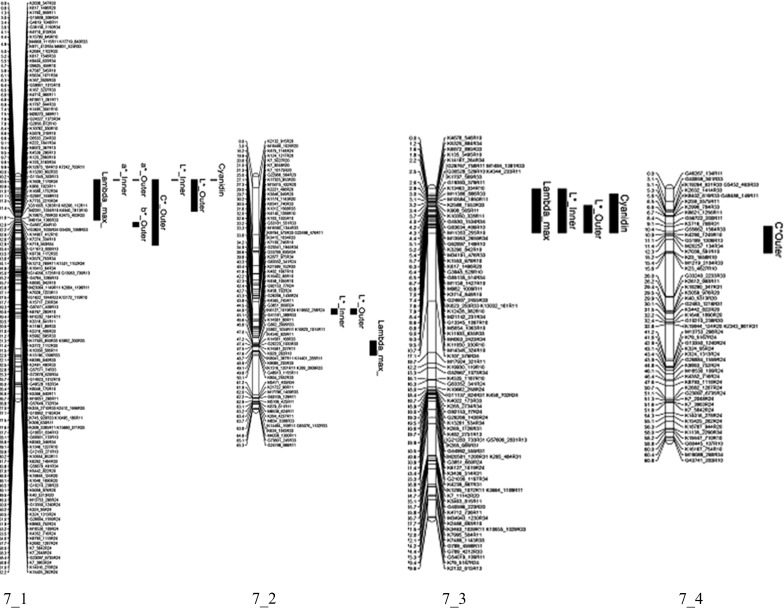
Four homolog linkage groups of ICM 7 of the genetic map of parent P540. The positions of the QTLs are represented by *black bars* (the *bar* presents the *lower* and *upper bounds* of the QTL)

QTLs were found for 10 of the 14 color traits measured in the K5 population (Table 2). Across the traits, ten QTLs were identified. Significant markers were found for all the color traits apart from a* Outer, C* Inner and *h*° Outer. For the traits; b* Inner, b* Outer, C* Outer, *h*° Inner, L* Inner, L* Outer, and Cyanidin, we identified a QTL on ICM 6 homolog 1 of parent P540 (Fig. 2). For the traits L* Inner, L* Outer (the same marker was significant on both parents), we also observed an allelic QTL on homolog 2 of ICM 6 and homolog 3 of ICM 6 of parent P867. For the trait *λ*_max_ we observed an allelic QTL on homolog 2 of ICM 7. On ICM 7 we identified QTLs on four homologs (Fig. 3). On the parental map of P867 we identified QTLs on ICM 1 homolog 1 and ICM 6 homolog 2 and ICM 6 homolog 3. The identified QTLs had an explained variance ranging from 7.3 % for L* Inner on ICM7_2 of P540 to 61.0 % for *λ*_max_ on ICM 7_1 and 7_3 (Fig. 3).

**Table 2:** QTLs for flower color traits showing the trait means per dosage class (only those classes that were present)

Trait	Markers	P540	P867	Segtype^a^	P540_cM	P867_cM	Benjamini–Hochberg-adjusted *p* values	−log_10_(p) (QTL)	% Explvar	0	1	2	3	4
*λ*_max_	K15799_649R10	7_1		s11000	4.4		0.006	4.18	11.2	520.7	523.6			
	K617_1496R20	7_1/7_3		s14100	0.8/6.3		0.000	25.8	61	516.2	523.8	523.3		
	K804_2552R33	7_2	7_2	s00121	50.1	31.6	0.024	3.4	10.4			519.9	523.0	522.6
	G85135_514R34	7_3		s00011	6.5		0.002	5	15.4			524	523.8	520.4
a* Inner	K617_1496R20	7_1/7_3		s14100	0.8/6.3		0.001	6.97	21.4	48.5	67.2	69.8		
b* Inner	K1138_459R34	6_1		s00011	17.9		0.004	5.81	16.0				48.9	32.0
	K11831_1118R10	6_1		s11000	36.4		0.011	4.6	12.4	32.7	47.8			
b* Outer	K1138_459R34	6_1		s00011	17.9		0.000	7.35	20.5				43.0	27.0
C* Outer	K1138_459R34	6_1		s00011	17.9		0.018	3.54	9.2				73.6	63.1
	K7755_2216R34	7_1		s00011	11.3		0.013	4.66	12.6				75.1	63.0
	K617_1496R20	7_1/7_3		s14100	0.8/6.3		0.002	6.77	20.8	55.4	72.2	77.6		
	K5716_768R31	7_4		s01210	9.1		0.027	3.29	9.9		57.0	69.8	74.2	
*h*° Inner	K4194_998R34	6_1		s00011	12.5		0.032	4.94	13.5				36.0	25.9
L* Inner	K1879_881R24	2_3/2_4		s00141	18.1/32.1		0.002	4.45	13.8			70.7	55.2	71.7
	K15465_153R33	6_1	6_2	s00121	27.7	23.1	0.001	5	15.4			49.5	62.6	70.9
	K11831_1118R10	6_1		s11000	36.4		0.000	7.12	19.9	70.0	52.2			
	K9445_363R24	6_3		s00121	3.9		0.020	3.08	9.4			48.9	63.3	65.8
	K5187_1076R33	6_4		s00121	5.5		0.038	3.27	10.0			72.2	58.3	54.0
	K7441_226R33	6_4	6_3	s00121	20.6	23.7	0.021	3.05	9.1			71.1	57.2	55.5
	K617_1496R20	7_1/7_3		s14100	0.8/6.3		0.000	11.02	32.3	80.1	54.4	52.1		
	D33788_695R34	7_2		s00011	38.6		0.027	2.93	7.3				65.4	54.3
	G85135_514R34	7_3		s00011	6.5		0.000	7.27	20.4				50.2	68.3
	K9130_421R33		1_1	s00121		56.2	0.001	4.64	14.4			49.8	61.3	70.8
	D11815_630R33		6_2	s00121		0.2	0.001	4.84	14.9			50.4	62.4	71.5
L* Outer	K1879_881R24	2_3/2_4		s00141	18.1/32.1		0.005	4.31	13.3			73.3	61.1	73.5
	K77_4131R33	6_1	6_2	s00121	27.3	0.9	0.000	5.4	16.6			56.9	66.9	74
	K5746_934R10	6_1		s11000	36.6		0.000	6.99	19.5	72.3	58.8			
	K2961_986R20	6_3		s14100	50.0		0.040	2.68	8.0	57.2	65.1	72.3		
	K416_1186R33	6_4	6_3	s00121	14.0	25.7	0.018	3.24	9.8			72.8	64.1	58.8
Cyanidin	K5746_934R10	6_1		s11000	36.6		0.020	4.57	12.3	3.0	6.5			
	K617_1496R20	7_1/7_3		s14100	0.8/6.3		0.017	5.9	18.2	1.2	6.1	5.8		
Pelargonidin	K617_1496R20	7_1/7_3		s14100	0.8/6.3		0.036	4.11	12.68	2.29	0.49	0.13		

Linkage groups (of the ICM rose map) and homologs of each linkage group are indicated, as well as parental origin of the marker allele associated with the trait. The last two numbers in the marker name refer to the segregation type where 10 = simplex × nulliplex, 11 = simplex × simplex, 20 duplex × nulliplex or 34 = triplex × quadruplex

^a^Each position represents the dosage class from 0 to 4 with 4 representing the fifth position. The number is the expected portion that should have the dosage

From a preliminary analysis of the data and observed flower color, we were able to separate the flower colors into four groups: dark red, red, pink and salmon. From each color class, we evaluated all genotypes and analyzed the relationship between the significant markers and the observed color attributes (Table 3). For the dark red color, the markers on ICM 2_3, 6_1, 6_3, 7_3 and 7_4 from parent P540 and 1_1 and 6_2 from parent P867 were present (i.e., dosage 1). For the red flowers, the markers on ICM 6_1 and 7_4 from parent P540 were present and the marker 6_2 from parent P867. Markers from ICM 7_1 and 7_3 from parent P540 were absent (i.e., dosage 0). For the pink flowers, the main markers present were from ICM 6_4, 7_2 and 7_4 from parent P540 and 6_3 and 7_2 from parent P867. The salmon flowers had markers from ICM 7_4 on parent P540 and 4_2 and 6_2 from parent P867. Interestingly the markers from ICM 6_1, 7_1 and 7_3 of parent P540 were absent in a majority of the salmon colored progeny and those from ICM 6_4, 7_2 and 7_4 from parent P540 were present.

**Table 3:** Significant markers associated with color mapped to the integrated consensus map ICM and respective homolog of the parents P540 and P867

Color	Code	K9130_421R33	K1879_881R24	D11815_630R33	K15465_153R33	K11831_1118R10	K2961_986R20	K9445_363R24
	P540		2_3		6_1	6_1	6_3	6_3
	cM Parent		18.1		27.7	36.4	50	3.9
	P540							
	cM Parent							
	P867	1_1		6_2	6_2			
	cM Parent	56.2		0.2	23.1			
Dark red	K121	1	1	1	1	1	0	1
Dark red	K165	1	1	1	1	1	0	1
Dark red	K167	1	1	1	1	0	1	1
Dark red	K210	1	1	1	1	1	0	1
Dark red	K229	1	1	1	1	1	1	1
Dark red	P540	1	1	1	1	1	1	1
Red	K026	1	1	1	1	1	1	1
Red	K069	0	1	1	1	0	1	1
Red	K126	1	1	1	1	1	1	1
Red	K148	1	1	1	1	1	0	
Red	K192	1	1	1	1	1	1	1
Red	K200	1	0	1	1	1	1	1
Pink	K001	1	1	1	1	0	1	1
Pink	K017	1	0	1	1	0	1	0
Pink	K098	0	0	0	0	0	1	0
Pink	K152	0	1	0	0	0	1	1
Pink	K171	0	1	0	0	0	1	0
Pink	K182	0	1	0	0	0	1	0
Salmon	P867	1	0	1	1	0	0	0
Salmon	K073	0	0	1	1	0	1	1
Salmon	K104	0	1	1	1	0	1	1
Salmon	K134	0	1	1	1	0	1	0
Salmon	K138	1	0	1	1	0	1	1
Salmon	K144	0	0	0	0	0	1	0
Salmon	K207	1	1	0	0	0	1	0

Color	Code	K416_1186R33	K5187_1076R33	K15799_649R10	K617_1496R20	K804_2552R33	G85135_514R34	K5716_768R31
	P540	6_4	6_4	7_1	7_1	7_2	7_3	7_4
	cM Parent	14.1	5.5	4.4	0.8	50.1	6.5	9.1
	P540				7_3			
	cM Parent				6.3			
	P867	6_3				7_2		
	cM Parent	25.7				31.6		
Dark red	K121	0	0	0	1	1	1	1
Dark red	K165	1	1	0	1	1	1	1
Dark red	K167	1	1	1	1	1	1	1
Dark red	K210	0	0	0	1	1	1	1
Dark red	K229	1	1	0	1	1	1	1
Dark red	P540	1	1	1	1	1	1	1
Red	K026	1	1	0	0	1	0	1
Red	K069	0	0	0	0	1	0	1
Red	K126	1	1	1	1	1	0	1
Red	K148	0	0	0	0	1	0	1
Red	K192	1	1	0	0	0	0	1
Red	K200	0	0	0	0	1	0	1
Pink	K001	1	1	0	1	1	1	1
Pink	K017	1	1	1	1	1	0	1
Pink	K098	1	1	1	1	1	0	1
Pink	K152	1	1	0	1	1	1	1
Pink	K171	1	1	0	0	1	0	1
Pink	K182	1	1	0	1	1	1	1
Salmon	P867	1	1	0	0	1	0	1
Salmon	K073	1	1	0	0	1	0	1
Salmon	K104	1	1	0	0	1	0	1
Salmon	K134	1	1	0	0	1	0	1
Salmon	K138	1	1	0	0	1	0	1
Salmon	K144	1	1	0	0	1	0	1
Salmon	K207	1	1	0	0	1	0	1

## Discussion

The range of colors from dark red to very pale salmon in the K5 population provided a good source of material to study the inheritance of color and anthocyanidins within a tetraploid population. Previously, there have been a number of studies on diploid populations (De Vries et al. 1980b; Debener 1999; Henz et al. 2015).

Within our tetraploid population, we detected QTLs on ICM 1, 2, 6 and 7. Previously, Debener (1999) mapped the gene for pink flower color *Blfa* (flower color) on ICM 2 and later on Henz et al. (2015) mapped major QTLs on ICM 2, 3, 4 and 6. The QTL on ICM 6 was at the center of ICM 6 in the region where a marker derived from a bHLH (basis helix-loop-helix) gene was mapped by Henz et al. (2015). They also mapped other genes involved in the flavonoid pathway on ICM 1, 3, 4, 6 and 7. Based on the reported marker positions of these genes, we hypothesize that the QTL we observed on ICM 6 is within the map region of the bHLH gene, the QTL on ICM 7 is in the region of DFR and the QTL on ICM 1 is within the region of GT-5 (glycosyltransferase). The bHLH genes are transcription factors involved in regulating many processes, including anthocyanin biosynthesis in plants. Within the K5 population, we had varying concentrations of cyanidin, consistent with the hypothesis that the QTL on ICM 6 contains the bHLH gene. The allelic variation of this bHLH gene could be responsible for the effect on the cyanidin content that would explain why, within our population within the four color classes, the QTL from ICM 6 was present. The marker on ICM 6 from the salmon father was present in all the genotypes that had the very light color and low levels of cyanidin, while the dark red, red and pink flowers had the marker from ICM 6 of the red mother. In different marker combinations, the ICM 6 marker was found to be significantly associated with the different amounts of total cyanidin present. Bushakra et al. (2013) showed that in raspberry a bHLH gene is involved in the sugar modification of cyanidin, rather than the production of total anthocyanins. However, in *Petunia* bHLH proteins have been shown to activate the transcription of flavonoid biosynthesis genes in the limbs of the petals and anthers (Koes et al. 2005). In morning glory, the lack of some bHLH gene expression resulted in a partial reduction in the expression of all anthocyanin biosynthesis genes (Park et al. 2007). Also in grape and *Litchi chinensis,* an important role for bHLH genes in anthocyanin biosynthesis has been found (Matus et al. 2010; Lai et al. 2016).

The light colored parent P867 contained low levels of pelargonidin and only traces of cyanidin, while the dark red colored parent P540 contained high levels of cyanidin, a relatively high level of the unidentified anthocyanidin but no pelargonidin.

The difference between the dark reds and the reds was that, the dark reds needed markers present in ICM 7_3 present while the red ones lacked ICM 7_3. This was an illustration on how the allelic variation even at a single locus has an effect on flower color. For the very light flowers (salmon types), we observed that the markers that are localized in the same position as the DFR copy found in ICM 7 of Henz et al. (2015) and on ICM 7_1 and ICM 7_3 in this study, were absent. Nakatsuka et al. (2007) showed that the manipulation of FLS and DFR, which are competing steps for flux toward flavonols and anthocyanins, resulted in a decrease or increase, respectively, in anthocyanin content in the flower.

With the data presented here, we have taken a step further in understanding the genetic control of anthocyanin biosynthesis and color development in tetraploid rose. This will aid in cultivar improvement by the ability to develop molecular markers for these traits. The ability to determine the alleles of the genes present can be a tool to be used by the breeder to determine in this case the shade of red or pink that is desirable. Red is by far the most important color in the market but still the most difficult to select for due to the various shades available. We have shown in this paper that to get the brighter red, one of the parents needs to have the markers for the genes that code for the expression of pelargonidin production.

## Electronic supplementary material

Below is the link to the electronic supplementary material.

Supplementary material 1 (DOCX 230 kb)

Supplementary material 2 (DOCX 28 kb)
