# Supplementary material for: Inheritance and QTL analysis of the determinants of flower color in tetraploid cut roses
Source: Mol Breed. 2016 Oct 7;36(10):143. doi: 10.1007/s11032-016-0565-9 (PMC5055553; doi:10.1007/s11032-016-0565-9)
Supplement: Supplementary file 1 — Supplementary material 1 (DOCX 230 kb) [file 11032_2016_565_MOESM1_ESM.docx]

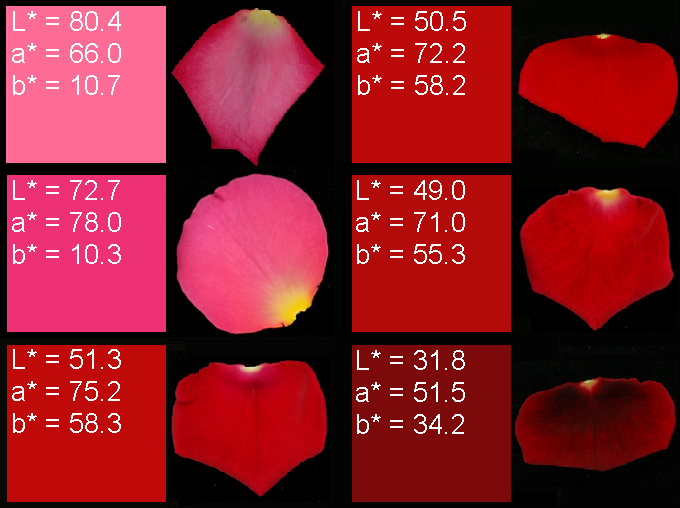


**Supplementary Fig. 1.** The L*, a* and b* coordinates that were calculated at a single spot on the petal (indicated by the arrow). Using these indices we calculated color parameters that were in good agreement with the visually observed color of the petal.
