# Supplementary material for: Inheritance and QTL analysis of the determinants of flower color in tetraploid cut roses
Source: Mol Breed. 2016 Oct 7;36(10):143. doi: 10.1007/s11032-016-0565-9 (PMC5055553; doi:10.1007/s11032-016-0565-9)
Supplement: Supplementary file 2 — Supplementary material 2 (DOCX 28 kb) [file 11032_2016_565_MOESM2_ESM.docx]

**Supplementary Table 1** Pearson correlation coefficients among the color traits.

**Correlation is significant at α=0.001; *Correlation is significant at α= 0.01

| **C* Inner** | -0.01 |  |  |  |  |  |  |  |  |  |  |  |  |
| --- | --- | --- | --- | --- | --- | --- | --- | --- | --- | --- | --- | --- | --- |
| **C*Outer** | 0.20* | 0.84** |  |  |  |  |  |  |  |  |  |  |  |
| **Cyanidin** | 0.43** | -0.05 | 0.16 |  |  |  |  |  |  |  |  |  |  |
| **hᴼ Inner** | -0.38** | 0.30** | 0.19* | 0.12 |  |  |  |  |  |  |  |  |  |
| **hᴼ Outer** | -0.08 | 0.13 | 0.25* | 0.51** | 0.73** |  |  |  |  |  |  |  |  |
| **L* Inner** | -0.50** | -0.18* | -0.38** | -0.87** | -0.16 | -0.51** |  |  |  |  |  |  |  |
| **L* Outer** | -0.54** | -0.29** | -0.45** | -0.81** | -0.17 | -0.50** | 0.94** |  |  |  |  |  |  |
| **Pelargonidin** | -0.59** | 0.40** | 0.23* | -0.08 | 0.38** | 0.19* | 0.06 | 0.03 |  |  |  |  |  |
| **Unidentified** | 0.33** | -0.06 | 0.12 | 0.87** | 0.1 | 0.45** | -0.71** | -0.64** | -0.05 |  |  |  |  |
| **a* Inner** | 0.23* | 0.91** | 0.80** | -0.05 | -0.1 | -0.15 | -0.18* | -0.29** | 0.18* | -0.06 |  |  |  |
| **a* Outer** | 0.24* | 0.79** | 0.86** | -0.11 | -0.15 | -0.26* | -0.12 | -0.20* | 0.13 | -0.12 | 0.89** |  |  |
| **b* Inner** | -0.27* | 0.80** | 0.62** | 0.04 | 0.80** | 0.53** | -0.19* | -0.27* | 0.52** | 0.03 | 0.48** | 0.38** |  |
| **b* Outer** | 0.01 | 0.50** | 0.68** | 0.44** | 0.65** | 0.86** | -0.53** | -0.56** | 0.28* | 0.39** | 0.26* | 0.22* | 0.72** |
|  | **λ_max_** | **C* Inner** | **C*Outer** | **Cyanidin** | **hᴼ Inner** | **hᴼ Outer** | **L* Inner** | **L* Outer** | **Pelargonidin** | **Unidentified** | **a* Inner** | **a* Outer** | **b* Inner** |
